# Vestigial-dependent induction contributes to robust patterning but is not essential for wing-fate recruitment in *Drosophila*

**DOI:** 10.1242/bio.059908

**Published:** 2023-05-18

**Authors:** Marycruz Flores-Flores, Luis Manuel Muñoz-Nava, Rafael Rodríguez-Muñoz, Jeremiah Zartman, Marcos Nahmad

**Affiliations:** ^1^ Department of Physiology, Biophysics, and Neurosciences, Centre for Research and Advanced Studies of the National Polytechnic Institute (Cinvestav-IPN), Mexico City 07360, Mexico; ^2^ Department of Chemical and Biomolecular Engineering, Notre Dame University, Notre Dame, IN 46556, USA

**Keywords:** Cell recruitment, Vestigial, *Drosophila* wing disc, Patterning, Robustness

## Abstract

Cell recruitment is a process by which a differentiated cell induces neighboring cells to adopt its same cell fate. In *Drosophila*, cells expressing the protein encoded by the wing selector gene, *vestigial* (*vg*), drive a feed-forward recruitment signal that expands the Vg pattern as a wave front. However, previous studies on Vg pattern formation do not reveal these dynamics. Here, we use live imaging to show that multiple cells at the periphery of the wing disc simultaneously activate a fluorescent reporter of the recruitment signal, suggesting that cells may be recruited without the need for their contact neighbors be recruited in advance. In support of this observation, when Vg expression is inhibited either at the dorsal–ventral boundary or away from it, the activation of the recruitment signal still occurs at a distance, suggesting that Vg expression is not absolutely required to send or propagate the recruitment signal. However, the strength and extent of the recruitment signal is clearly compromised. We conclude that a feed-forward, contact-dependent cell recruitment process is not essential for Vg patterning, but it is necessary for robustness. Overall, our findings reveal a previously unidentified role of cell recruitment as a robustness-conferring cell differentiation mechanism.

## Teaser

**Summary:** Vestigial (Vg) patterning establishes wing fate in the developing wing of Drosophila. This study shows that a Vg-independent signal may drive Vg expression, but the feed-forward Vg-dependent signal is necessary for pattern sharpness and robustness.

## INTRODUCTION

Developmental patterning is determined by cell-autonomous and non-cell-autonomous induction signals that establishes cell proliferation, differentiation, and morphogenesis (Perrimon et al., 2012). A particular case of induction is cell recruitment, in which a differentiated cell recruits its neighbors to differentiate into the same type as itself (Muñoz-Nava et al., 2021). Although cell recruitment is a widespread phenomenon in many developmental contexts such as the mammalian inner ear, thyroid, and kidney, and the *Drosophila* wing (Kiernan, 2013; Lindström et al., 2018; Nilsson and Fagman, 2017; Zecca and Struhl, 2007), the details about how recruitment signals regulate patterning and growth of populations of differentiated cells remains largely unknown. Moreover, the objective of cell recruitment as a developmental mechanism remains unclear. Is there a particular advantage of cell recruitment over other induction mechanisms? Particularly, why is cell recruitment used as a patterning mechanism when, in principle, the same output may be achieved through classical morphogen patterning?

During the development of imaginal discs in *Drosophila*, wing fate is specified by the expression of the selector gene, *vestigial* (*vg*). *vg* knockout results in loss of wing structure (Williams et al., 1991, 1993) whereas its overexpression in other imaginal discs induces their transformation into wing-like tissue (Baena-López and García-Bellido, 2003; Kim et al., 1996). Vg patterning is a complex process that requires the integration of several signaling pathways. First, the morphogens that pattern the orthogonal axes of the wing, Wingless (Wg) and Decapentaplegic (Dpp), that emanate from the dorsal–ventral (DV) and anterior–posterior (AP) boundaries, respectively, establish a region of competence for *vg* expression and wing-fate differentiation (Couso et al., 1994; Kim et al., 1996; Klein and Arias, 1998; Neumann and Cohen, 1996; Williams et al., 1994). Cells at the DV boundary express *vg* through the boundary enhancer *vg*^BE^ in response to Notch signaling (Irvine and Vogt, 1997; Kim et al., 1996; Williams et al., 1994). Then, the Vg pattern presumably expands by proliferation of Vg-expressing cells and through a feed-forward cell recruitment signal in which Vg-expressing cells that receive Wg and Dpp signaling induce neighboring undifferentiated cells to express *vg* and establish the wing fate (Zecca and Struhl, 2007, 2021). The molecular identity of this cell recruitment signal is the polarization of two protocadherins, Fat (Ft) and Dachsous (Ds), which interact in a heterotypical manner at the plasma membranes of adjacent cells (Zecca and Struhl, 2010). In early wing disc development, Ft-Ds complexes are uniform and randomly distributed throughout the tissue. However, Vg initiates the recruitment signal by inhibiting *ds* transcription, thereby creating asymmetry in Ft-Ds localization. This polarization leads to the inactivation of the Hippo pathway in the neighboring cells, thereby importing Yorkie (Yki) to the nucleus and permitting its transcriptional activity on the *vg* quadrant enhancer *vg*^QE^ (Zecca and Struhl, 2010; Goulev et al., 2008). This feed-forward system is expected to self-propagate until no competent cells (i.e. cells that receive Wg or Dpp signaling) are available for recruitment.

**Fig. 1.**
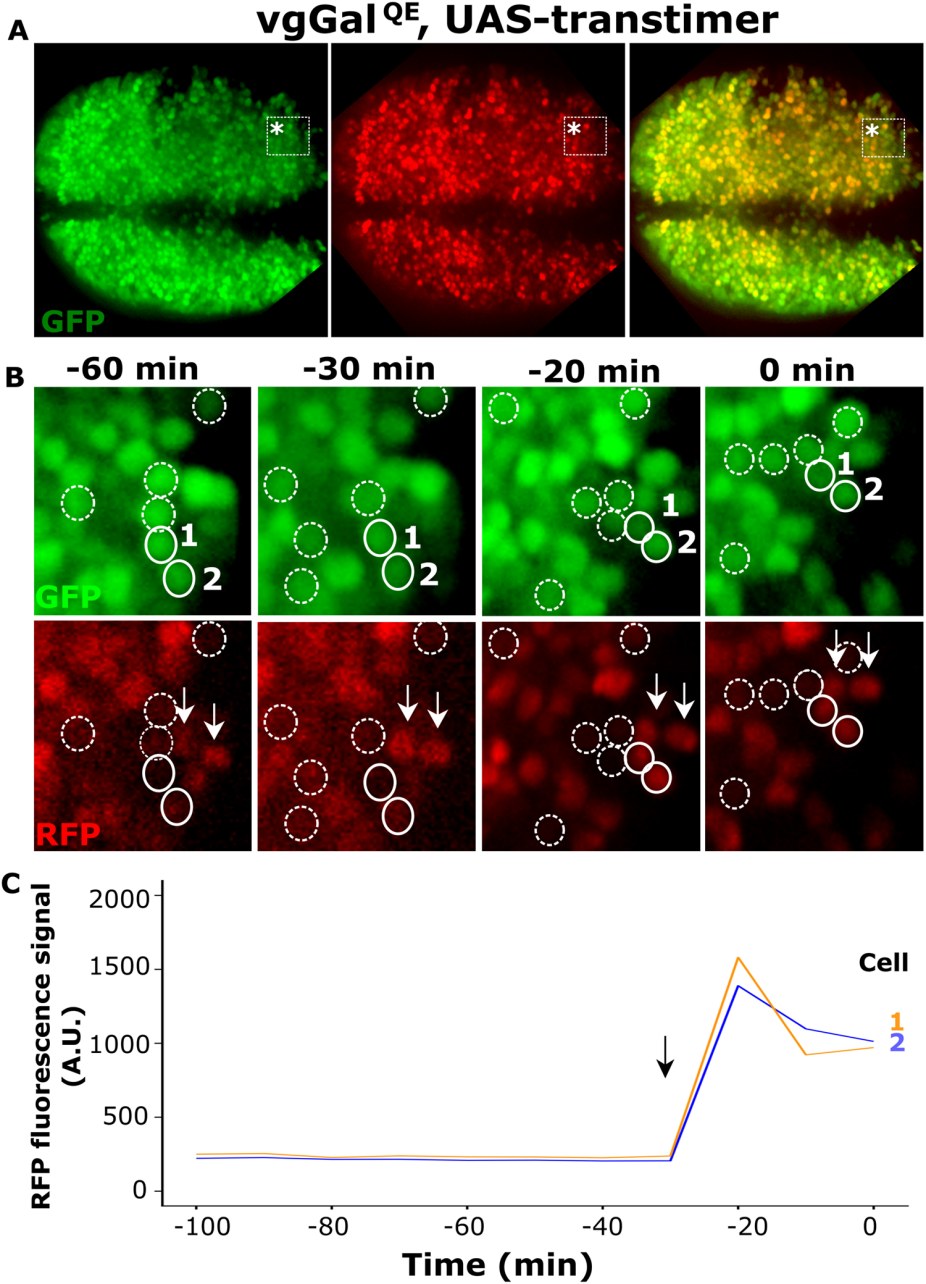
**Live imaging of cell recruitment in the Drosophila wing disc.** (A) Sample z-stack snapshot of an *ex-vivo* cultured third-instar wing disc expressing the Transtimer reporter under *vg*^QE^-Gal4 control. The tissue was imaged live in Grace's medium for ∼8 h every 10 min using a microfluidic device under constant flux. (B) Enlargement of the region depicted in the dotted rectangle marked with an asterisk; GFP and RFP channels are displayed separately. The last time-point of the movie was set as 0 min and the dynamics of expression at three prior time-points (–20, −30, and −60 min) are displayed. We manually circled cells that express GFP but not RFP (dotted circles) and cells that turned on RFP (solid circles) within these times. Notice that cells labeled 1 and 2 turned RFP on between the −30 and −20 min time points. Cells marked by arrows are used only as a reference. (C) RFP fluorescence intensity within cells one and two in the last 100 min of the movie.

**Fig. 2.**
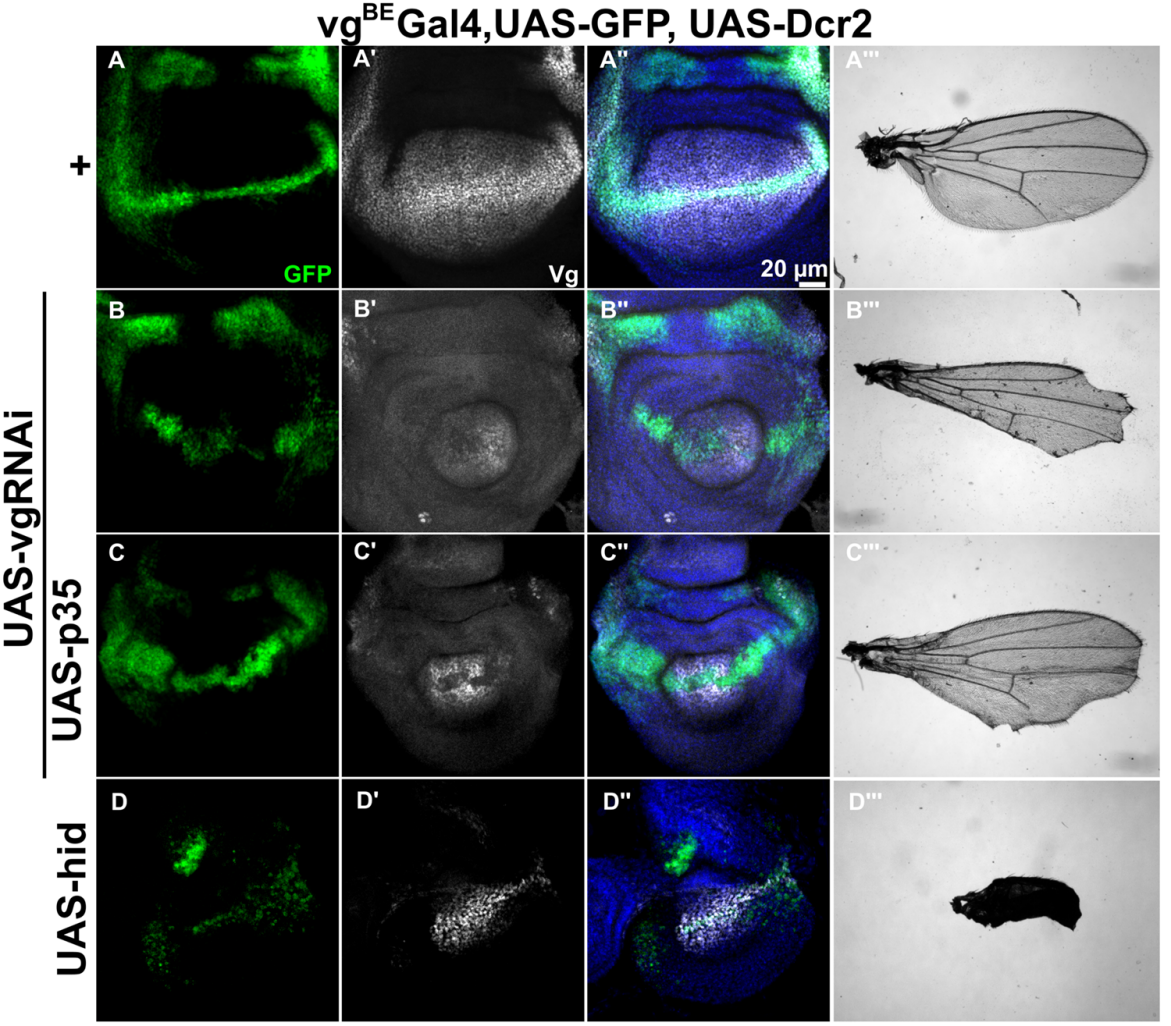
**Inhibition of Vg at the DV boundary affect cell survival and growth of the wing pouch and adult wing, but is not required for long-range Vg induction.** (A-D, A′-D′, A″-D″) Representative images of third-instar wing imaginal discs stained with GFP (green), Vg (white, using a Vg antibody), and DAPI (blue) that ectopically express at the DV boundary (*vg*^BE^ domain using the Gal4-UAS system): GFP and Dcr2 (A-A″); GFP, Dcr2, and *vg*RNAi (B-B″); GFP, Dcr2, *vg*RNAi, and p35 (C-C″); or hid (D-D″). In all images, anterior is to the left and dorsal is up. (A‴-D‴) Representative adult wings that result from the corresponding genotypes.

**Fig. 3.**
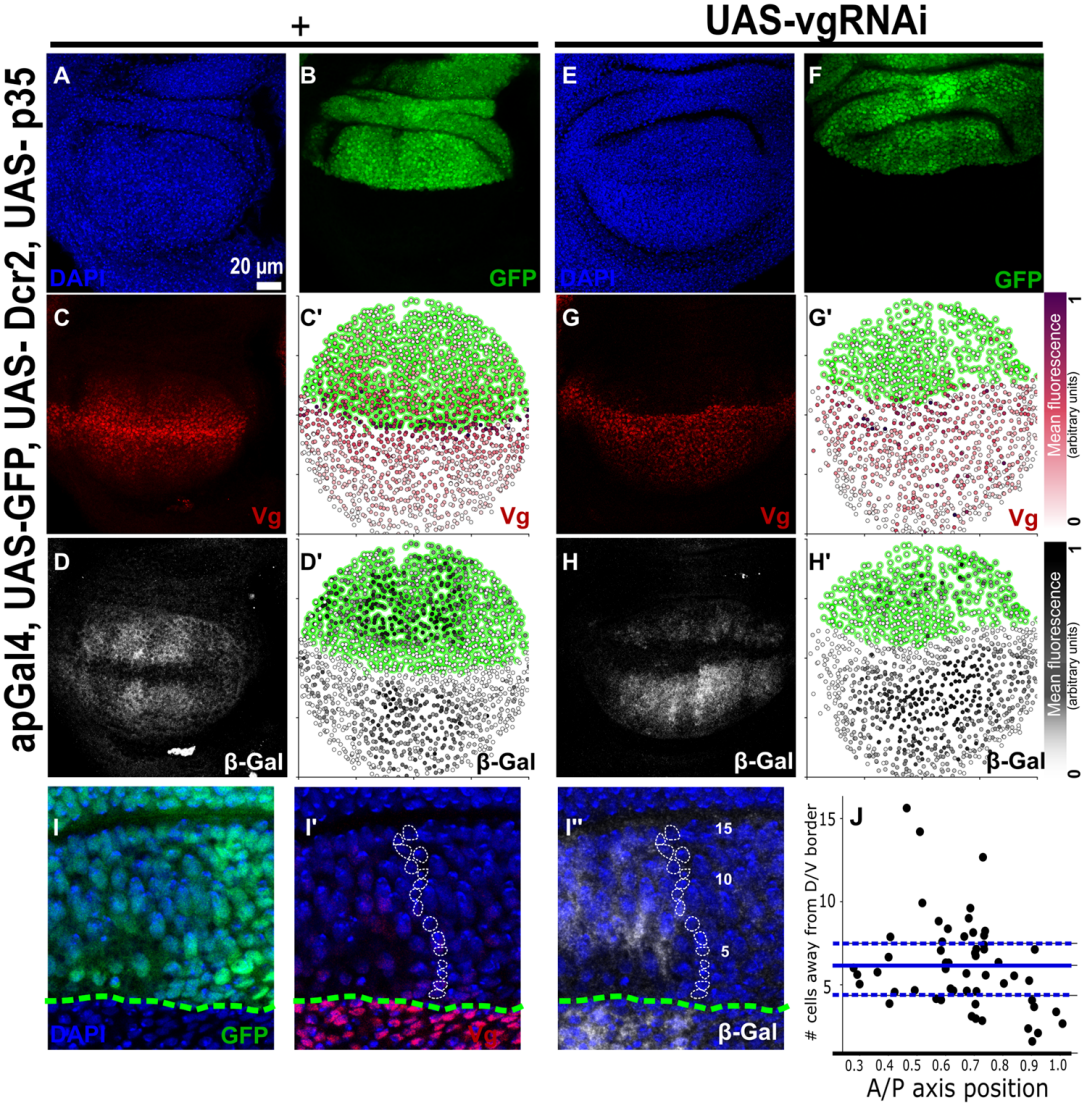
**Cell recruitment signal can propagate throughout the wing pouch without a Vg-dependent feed-forward mechanism.** (A-H′) Representative images of third instar wing imaginal discs stained with DAPI (A,E), GFP (B,F), Vestigial (C,G), and β-Gal (D,H) antibodies, either in a wild-type (A-D) or a Vg-knockdown (E-H) background in the dorsal compartment. (C′,G′,D′,H′) Qualitative representation of nuclear Vg or cytoplasmic β-Gal fluorescence signal, respectively. (I-I″) Representative enlargement view depicting a representative disc of the same genotype as (E-H) showing a path of cells from the DV border (marked with DAPI expression in blue) to illustrate the range of the recruitment signal (I″, delineated in white). The DV border (bottom green-dotted line) was determined using GFP expression (I). (J′) Quantification of number of cells from the DV border that express at least half-maximal levels of β-Gal. Blue line shows the mean; Q1 and Q3 are represented by blue dotted lines). Control discs (*n*=9); *vg*RNAi knockdown discs (*n*=6).

**Fig. 4.**
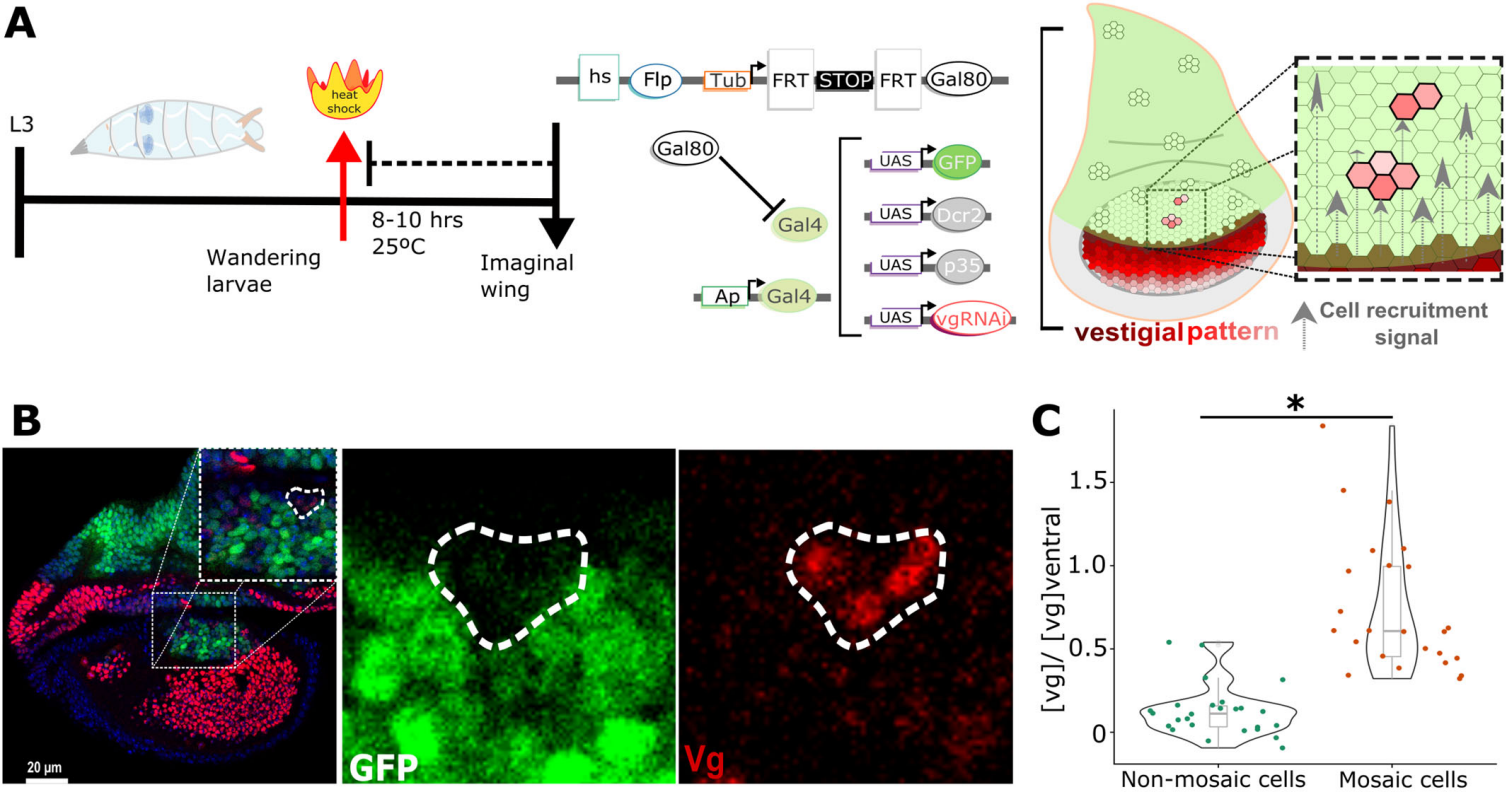
**The cell recruitment signal activates Vg expression at a distance.** (A) Experimental design for the generation of Gal80-positive clones using a heat-shock Flp-mediated recombination in *ap*Gal4-UAS-GFP, p35, Dcr2, *vg*RNAi wing discs (vg-pattern, red pattern). Mid-third instar larvae were heat shocked for 10 min at 38°C (red arrow) and fixed/dissected 8-10 h later (late-third instar, black arrow). Before heat-shock treatment, an FRT-STOP-FRT cassette prevents Gal80 from being expressed ubiquitously (under the regulation of a *tubulin* promoter). However, after heat shock, the Flp recombinase will be able to remove the FRT-STOP-FRT cassette in some cells, creating mosaics of Gal80 expressing cells. If a mosaic is located into the dorsal compartment (white clones in the disc cartoon), it prevents Gal4 from activating expression of GFP, p35, Dcr2, and *vg*RNAi (p35 and Dcr2 are expressed to prevent cell death and potentiate the RNAi effect, respectively). (B) Images of wing discs of the experimental design shown in A, stained with a Vg antibody (red), GPF (green) and DAPI (blue). Inset show the magnification of an area depicting a mosaic completely surrounded by GFP-expressing cells. (C) Quantification of cells located within and outside of the mosaic normalized by the intensity of Vg at the same position in the ventral compartment.

The feed-forward model of Vg-dependent recruitment suggests that the Vg pattern propagates as a wave front, in which newly recruited cells acquire a certain level of Vg expression (enough to drive wing fate differentiation), before passing the recruitment signal to the following cell in a contact-dependent manner (Zecca and Struhl, 2010). However, prior studies examining the dynamics of Vg patterning show a gradient of Vg expression (Baena-López and García-Bellido, 2003; Muñoz-Nava et al., 2020), which are not consistent with a wave-front mode of propagation. In order to resolve this discrepancy, here, we examined the dynamics of cell recruitment in the developing wing imaginal disc using live imaging of a dual fluorescent reporter system. We observe the presence of neighboring cells that simultaneously received the recruitment signal and we failed to observe a defined front of recruitment (Fig. 1). This experiment confirmed prior fixed tissue experiments (Muñoz-Nava et al., 2020), and suggested that signal-relay expansion of Vg expression is not required for the propagation of recruitment. To test this prediction, we first tested the requirement of a source of Vg-expressing cells by knocking-down Vg expression at cells of the DV boundary and found that it affects cell survival, but when these cells were rescued from cell death, a Vg-independent signal is capable of driving Vg expression in a nearly normal pattern, although the wing pouch and the resulting adult wings are significantly diminished in size (Fig. 2). Furthermore, when Vg expression was knocked down in the whole dorsal compartment (to levels that do not allow Vg function), *vg*^QE^ expression still propagates nearly as far as in wild-type discs, although at reduced expression levels (Fig. 3). This reveals that a signal that activates the *vg*^QE^ may induce Vg expression at a distance independently of a contact-dependent cell recruitment signal (Fig. 4). Taken together, we propose that a feed-forward Vg patterning is established by two signaling mechanisms: Vg-independent induction signal that likely provides Vg patterning its graded nature; and a feed-forward Vg-dependent recruitment signal, as previously proposed by Zecca and Struhl that promotes robustness of the final Vg pattern.

## RESULTS

### Live imaging reveals simultaneous expression of the recruitment signal in neighboring cells

Previous data have revealed the participation of cell recruitment in Vg patterning and wing growth (Muñoz-Nava et al., 2020; Zecca and Struhl, 2007). However, fixed-tissue data do not reveal the actual dynamics of recruitment signal propagation. To investigate if sequential Vg expression is necessary for recruitment propagation in a signal relay, we used live imaging of a late third-instar wing disc cultured *ex vivo* that expresses a dual-color fluorescent reporter, known as Transtimer; which produces a rapid, unstable GFP and a slow, stable RFP reporter (He et al., 2019). By driving the Transtimer reporter under *vg*^QE^-Gal4 control, we were able to distinguish yellow (RFP+GFP) cells that established *vg*^QE^ expression several hours ago from green (GFP only) cells that were newly recruited (Fig. 1A). Indeed, while most cells were expressing both reporters (yellow), we observe several green cells at the edges of the Vg pattern (Fig. 1B; dotted circles). These green cells, which received the recruitment signal in the past few hours, are not located in a specific spatial pattern at the boundary of the Vg-expressing domain, suggesting against a wave-front mode of propagation. This observation is consistent with a previous result in fixed tissues (Muñoz-Nava et al., 2020), but provides an additional insight. By examining the last time-point of the movie and tracking cells backwards, we were able to identify cells that turn from green into yellow, thus providing evidence that these cells sustain expression through the *vg*^QE^ (Fig. 1B; solid circles). Furthermore, we identified neighboring cells that simultaneously turned into yellow cells (Fig. 1C; cells 1 and 2). While one of these cells (labeled as cell 1) is in direct contact with another yellow cell, the other (labeled as cell 2) does not appear to be in direct contact with any other yellow cell, suggesting that the propagation of the recruitment signal without a Vg-dependent feed-forward signal relay is feasible. This experiment provides the first live-imaging evidence that the recruitment signaling may propagate in a Vg-independent manner.

### Vg expression at the DV boundary is required for survival of inducer cells, but is not essential for long-range induction of Vg

We then asked if Vg initiates recruitment from the DV boundary, i.e., if Vg expression at the DV boundary was necessary to propagate its own expression in the rest of the wing pouch (Fig. 2A′,A″). Since Vg is expressed at the DV boundary through the *vg*^BE^ (Fig. 2A; Kim et al., 1996; Williams et al., 1994), we expressed a *vg*RNAi construct [potentiated by Dicer2 (Dcr2)] under a *vg*^BE^-Gal4 driver to downregulate Vg expression at the DV boundary (marked with a UAS-GFP reporter). We found that expression of the *vg*RNAi not only strongly reduced Vg expression at the DV boundary, but also affected Vg expression in the rest of the wing pouch (compare Fig. 2A′ to B′). We also observed that the size of the wing pouch area was dramatically reduced (compare Fig. 2A″ to B″) and much of the distal adult wing (including the wing margin) was missing (compare Fig. 2A‴ to B‴). While this result supports a role for Vg in recruiting and promoting growth in the rest of the tissue, we also noticed in this experiment that the GFP reporter was mostly missing from the DV boundary within the wing pouch (compare Fig. 2A to B). This suggests that these cells undergo apoptosis without Vg expression, in agreement with previous studies (Baena-Lopez and García-Bellido, 2006; Delanoue et al., 2004). Indeed, when survival of these cells is ensured by co-expressing the anti-apoptotic protein p35, the GFP reporter expression along the DV boundary is recovered (Fig. 2C) and high level of Vg expression is observed in the wing pouch, except at the DV boundary itself as expected from to the ectopic expression of the *vg*RNAi (Fig. 2C′). Remarkably however, despite the rescue of GFP and Vg expression within and away from the DV boundary, respectively, the size of the wing pouch and adult wing remains strongly reduced (Fig. 2C″,C‴). This result confirms that Vg expression at the DV boundary is needed for cell survival and tissue growth (Delanoue et al., 2004; Pérez et al., 2011), but not for Vg induction away from the DV boundary. In fact, this interpretation is further supported by an experiment in which cells at the DV boundary are eliminated by the overexpression of *hid* that triggers apoptosis (Fig. 2D). In this case, the pattern of Vg in the wing pouch is dramatically affected (Fig. 2D′) and the resulting adult wing resembles a *vg* mutant (Williams et al., 1993; Fig. 2D‴). Taken together, we conclude that cells at the DV boundary depend on Vg to survive and proliferate, but not for Vg induction in the rest of the pouch, arguing against the hypothesis that Vg patterning is initiated by cell recruitment with a source of Vg at the DV boundary.

### Vg knockdown in the dorsal compartment does not prevent the activation of the *vg*^QE^

In the previous experiment, we showed that Vg expression at the DV boundary is dispensable for propagation of the Vg pattern throughout the wing pouch. We next tested whether Vg is needed for feed-forward propagation of the recruitment signal away from the DV boundary. Since the recruitment signal works through the activation of the *vg*^QE^ (Zecca and Struhl, 2007, 2010), we investigated if propagation of a *vg*^QE^lacZ reporter depends on Vg expression. With this aim, we expressed a *vg*RNAi under *apterous* (*ap*)-Gal4 control, which knocks Vg expression down in all the cells of the dorsal compartment (marked with a GFP reporter, Fig. 3A-D). To potentiate the effect of the RNAi and to avoid cell death resulting from the lack of Vg, we also co-expressed Dcr2 and p35, respectively. As expected, Vg is nearly eliminated in all cells of the dorsal compartment (compare Fig. 3G,G′ to C,C′). In contrast, the *vg*^QE^lacZ reporter was clearly detected (to at least half of its maximum) in a broad domain in the dorsal compartment (Fig. 3H,H′), reaching up to 15 cells away from the DV boundary (Fig. 3I-I″,J′). However, β-Gal expression levels resulting from the *vg*^QE^lacZ reporter in Vg- knockdown discs were significantly lower and did not pattern the dorsal region of the wing pouch to the same extent as in control discs (compare Fig. 3D to H). Since β-Gal expression levels were lower than normal in Vg-knockdown discs, we also considered the possibility that β-Gal expression resulted from Vg levels that remained after Vg knockdown. To evaluate this possibility, we quantified nuclear Vg and cytoplasmic β-Gal expression (Fig. S1), and compared the distributions of the Vg to β-Gal ratios in the dorsal compartment of Vg- knockdown and control discs. We found that residual levels of Vg remaining from *vg*RNAi expression do not explain the distribution of β-Gal expression (Fig. S2). These results strongly suggest that *vg* patterning is initiated or is partially driven by an induction signal independently of a Vg feed-forward recruitment signal.

### A Vg-independent cell recruitment signal is capable to activate Vg expression

Our previous experiment shows that *vg*^QE^lacZ expression can be detected several cells away from the DV boundary in a Vg-independent way, but the reporter expression is reduced compared to a wild-type control (Fig. 3). Therefore, it remains unclear whether this Vg-independent signal is sufficient to activate and sustain Vg expression. To test this, we used the FLP-FRT system to generate small mosaics expressing Gal80, which inactivates the Gal4 system (McGuire et al., 2003). When cells within these mosaics fall within the dorsal compartment, but away from the DV boundary, they behave as wild type cells (GFP-) surrounded by cells that express GFP, *vg*RNAi, p35, and Dcr2 under the control of *ap-*Gal4 (Fig. 4A). As in our prior experiment, GFP+ cells in the dorsal compartment are unable to propagate a Vg-dependent feed-forward recruitment signal, but GFP− cells within Gal80-expressing mosaics when surrounded by GFP+ cells will express Vg only through the Vg-independent induction signal. Indeed, we found that Vg is expressed in isolated Gal80-expressing clones within the dorsal compartment away from the DV boundary (Fig. 4B), suggesting that the recruitment signal is sufficient to drive Vg expression without a Vg-dependent signal relay. In fact, a quantification of this experiment shows that Vg levels within isolated Gal80+ clones are significantly higher than in neighboring cells outside of the mosaic and at least half the levels as in cells located at the same distance from the DV boundary in the control (ventral) compartment (Fig. 4C). We conclude that isolated GFP- mosaics may turn on Vg expression away from the DV boundary, even when they are not in direct contact with the source of Vg-expressing cells.

## DISCUSSION

What is the optimal mechanism to establish a pattern of cell differentiation during development? It depends on what is the objective. If the system prioritizes speed over precision or robustness then long-range diffusible or polarization signals are more appropriate than sequential rounds of contact-based induction. In the *Drosophila* wing disc, the *vg*^QE^ responds to long-range morphogens and polarization signals, but these have been proposed as a pre-requisite for the propagation of a cell-to-cell feed-forward mechanism in which cells need to express a certain level of Vg, before recruiting its immediate neighbors. Here we evaluate whether this Vg-dependent signal relay mechanism is absolutely required to propagate Vg expression at the distance. Our results argue against this hypothesis and challenges the idea that Vg expression propagates as a signal-relay process, in which cells become recruiters only when they receive enough Vg to acquire the wing fate themselves (Zecca and Struhl, 2010). Are the low, residual levels of Vg that remain from *vg*RNAi repression sufficient to drive Vg-mediated wing-fate differentiation? Our experiments argue against this possibility. For instance, we found that when we expressed *vg*RNAi in the dorsal compartment (as in Figs 3 and 4), the expression of DSRF, the product of the gene *blistered* that is downstream of Vg in the wing-differentiation pathway is missing (Fig. S3). In addition, when *vg*RNAi is expressed in the posterior compartment using the *engrailed*-Gal4 driver, the posterior portion of the adult wing is missing (Fig. S4). We conclude that Vg patterning may be established to some extent in the absence of a contact-dependent Vg feed-forward signal.

Prior work supports that Wg and Dpp emanating from the DV and AP boundaries, respectively, are necessary for Vg expression (Zecca and Struhl, 2007, 2010; 2021; Parker and Struhl, 2020). Are Wg and Dpp sufficient to drive the expression of the *vg*^QE^ in the absence of a Vg-dependent feed-forward signal? The answer is most likely no. First, because in these studies, Wg and Dpp can only drive expression of Vg on their own close to the compartment boundaries where signaling is very strong, or under conditions of Wg or Dpp overexpression (Parker and Struhl, 2020). In addition, a previous study identified that in wild-type conditions, the range of Wg signaling does not exceed 11 cells (Chaudhary et al., 2019), while we detect *vg*^QE^lacZ expression in some cells up to 15-cells away from the DV boundary (Fig. 3I-I″,J). We suggest instead that the Vg-independent activation of *vg*^QE^lacZ expression in our experiment is driven by Ft-Ds polarization. In fact, a prior computational study shows that Ft-Ds polarization may be achieved to some extent without Vg feed-forward propagation (Wortman et al., 2017). This Vg-independent recruitment propagation of Ft-Ds signaling may be responsible for the graded dynamics of the Vg pattern (Muñoz-Nava et al., 2020).

Our work demonstrates that the Vg pattern is established in two complementary ways. A Vg-independent signal that propagates quickly to initiate Vg expression and likely explains the graded nature of the pattern; and a feed-forward, Vg-dependent signal that elevates Vg levels and ensures a robust pattern throughout the wing pouch. Why does pattern formation in this system uses both mechanisms, when any of them could, in principle, support Vg patterning on their own? The Vg-independent mechanism offers speed, while the Vg-dependent feed-forward signal offers reliability. By combining both, the Vg-independent signal initiates patterning by ‘reserving’ a population of cells into the wing fate while maintaining them alive and actively proliferating, whereas the feed-forward mechanisms ensures that all cells attain sufficient levels of Vg expression to ‘complete’ the wing specification domain. Together, these signals offer a layered genetic architecture that ensures robust specification of cell fates, extending the repertoire of systems in which feed-forward loop regulatory networks provide robustness and canalization in developmental systems (Ducuing et al., 2015; Le and Kwon, 2013).

## MATERIALS AND METHODS

### *Drosophila* stocks and crosses

The following stocks and crosses were used:

Fig. 1: sco/SM5; *vg^QE^*Gal4 (BDSC #8229). UAS-Transtimer/SM5; TubGal80ts(BDSC #7017)/TM6B. The UAS-Transtimer was provided by Li He (Norbert Perrimon's Lab, Harvard Medical School, MA, USA).

Fig. 2. *vg^BE^*Gal4 (BDSC #8222)/SM5; UAS-GPFn (BDSC #4776)/TM6B. UAS-vgRNAi (2nd chr.,) from Vienna *Drosophila* Resource Center #16896)/SM5; UAS-Dcr2/TM6B. UAS-p35 (BDSC #6298); UAS-vgRNAi/SM5, UAS-Dcr2 (BDSC #90938)/TM6B. UAS-hid (BDSC #65408)/SM5; MKRS, hs-Flp/TM6B.

Fig. 3 and Fig. S3, apGal4 (BDSC #56807), UAS-GPFn (BDSC #4775)/SM5; vgQElacz (3nd chr.,)/TM6B. UAS-vgRNAi/SM5, UAS-Dcr2 (BDSC #90938)//TM6B.

Fig. 4, hs-Flp; apGal4 (BDSC #56807), UAS-GPFn (BDSC #4775)/SM5; Tub-FRT-STOP-FRT-Gal80 (BDSC #5145)/TM6B. UAS-p35 (BDSC #6298); UAS-vgRNAi/SM5, UAS-Dcr2 (BDSC #90938)//TM6B.

Unless indicated otherwise, all flies and crosses were reared on standard culture medium at 25°C.

### Heat-shock induction of flip-out clones

Marked clones were generated by Flp-mediated subjecting the early-third-instar larvae to a 38°C heat-shock for 10 min. Larvae were then shifted to 25°C and were dissected 8-10 h later for antibody staining.

### Live imagining

PETL (polyethylene terephthalate laminate)-based microfluidic devices were loaded with supplemented Grace's medium (Levis et al., 2019). Organs were placed on the outlet and drawn into the device by negative pressure while suspended in supplemented Grace's medium. Media flowing at 1 μl/h was used throughout imaging in the device.

We expressed the Transtimer reporter downstream the *vg*^QE^ using the *Drosophila* Gal4-UAS system. At the third-instar larval stage, we dissected the wing disc and loaded into the microfluidic chamber, using Grace's medium supplemented with Bis-Tris, Penn-Strp, and FBS (Dye et al., 2017).

Time-lapse confocal imaging was done using a Nikon Eclipse T*i* spinning disc confocal microscope (Andor). 40×/1.49-oil, and 100×/1.49-oil objectives was used for experiment. Snap-shoot was taken each 10 min.

Images were captured using MetaMorph software. Image processing was performed using Image J and imported into R for quantification.

### Immunostaining and microscopy

Wing imaginal discs were dissected from third-instar larvae of both sexes, unless indicated. For larva dissected at a specific age AEL. After dissection in a stereoscopic microscope (Nikon SMZ800), discs were fixed in PEM-T (PEM with 0.1% of Triton X-100) with 4% paraformaldehyde, washed three times and blocked in PEM-T with 0.5% of BSA (Bovine Serum Albumin) for 2 h at room temperature. Then, samples were stained with primary antibodies at 4°C overnight at the following dilutions: rabbit anti-Vg (a gift from Sean Carroll and Kristen Guss, 1:200), guinea pig anti-Vg (a gift from Gary Struhl, 1:200), mouse anti-beta Galactosidase (1:200). DAPI (1:1000) was used to stain nuclei. Primary antibodies were detected with Alexa Fluor 555 anti-mouse and Alexa Fluor 647 anti-guinea pig/anti-rabbit (1:1000). Imaging was done with a confocal microscope (Leica TCS SP8 Confocal Microscope) using a 63X oil-immersion objective.

### Image analysis and quantification

Wing imaginal discs were imaged as stated in Materials and Methods. We selected three representative confocal z-stack slices in each disc; z-slices are chosen far enough so that nuclei are not double-counted. Using Image J, we used the DAPI channel to set a threshold and create a binarized representation. We then segmented the binarized image by applying a watershed filter to obtain a nuclear segmentation mask. The image was exported to Matlab, where using the Particle Analysis function to remove particles (<6.8 pixels). To analyze the cytoplasmic *β*-Gal signal, we used the final nuclear segmentation mask data and calculated the distance between all the centroids to find the minimum distance. The regionprops function in Matlab was used to calculate the mean fluorescence intensity value of each nuclei or cytoplasmic region.

For the quantification of intensities in Fig. 4, we first obtained the pattern of Vg intensity as a function of distance from the dorsal to the ventral fold. Then, we plotted the average intensity values of Vg within the selected rectangle (30 pixels width), using the Image J Plot Profile function, against the distance between folds. Finally, we looked for the clones and measure the Vg intensity in each cell within and just outside the clone.

To normalize Vg levels, we imported both data sets in R Studio and subtracted background levels of Vg. Background levels were obtained averaging Vg levels inside a square (50×50px) in the notum region. We then divided each pixel of Vg intensity (to the maximum Vg value). Distance between folds was also normalized so that position was reported in relative units (0 and 1).

## Supplementary Material

10.1242/biolopen.059908_sup1 Supplementary information
