# Supplementary material for: Vestigial-dependent induction contributes to robust patterning but is not essential for wing-fate recruitment in Drosophila
Source: Biol Open. 2023 May 18;12(5):bio059908. doi: 10.1242/bio.059908 (PMC10214856; doi:10.1242/bio.059908)
Supplement: Supplementary information [file biolopen-12-059908-s1.pdf]

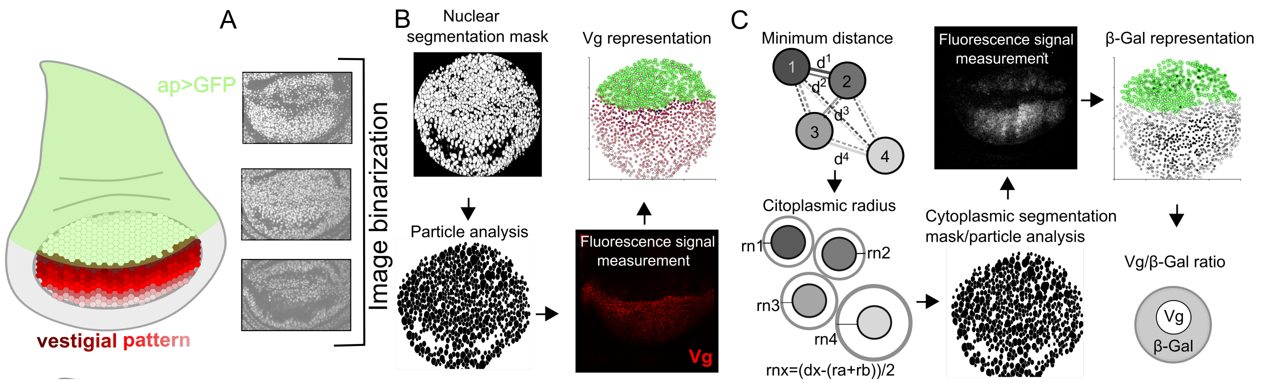

Fig. S1 (related to Fig. 3). Analysis of nuclear Vg and cytoplasmic β–Gal expression. (Left) Cartoon of the wing imaginal disc depicting the experimental design of Fig. 3; the *apterous*-Gal4 drives ectopic expression of GFP, Dcr2, p35, and *vg*RNAi in the dorsal compartment (green), whereas the Vg pattern remains unaffected in the ventral compartment (red). (A) Binarization of three representative confocal z-stack slices of a wing imaginal disc in the DAPI channel. (B) Nuclear segmentation mask was created using ImageJ for each z-stack slice. Expression that does not appear to mark nuclei were filtered out (using a threshold filter setting) and the Vg antibody signal inside each nucleus was analyzed using the Particle Analysis function in ImageJ (see Material and Methods). (C) Cytoplasmic regions were obtained as follows. First, distances between each neighboring nuclei centroids were obtained (top) and the minimum of these numbers was computed. Larger concentric circles were obtained using this minimal distance to estimate the cytoplasmic β-Gal fluorescence signal within a ring around each nucleus, using a cytoplasmic segmentation mask (see Material and Methods). The average Vg and β-Gal fluorescence levels within each nuclear and cytoplasmic area, respectively, are represented and obtained to compute the corresponding Vg to β-Gal ratios for each cell.

Biology Open • Supplementary information

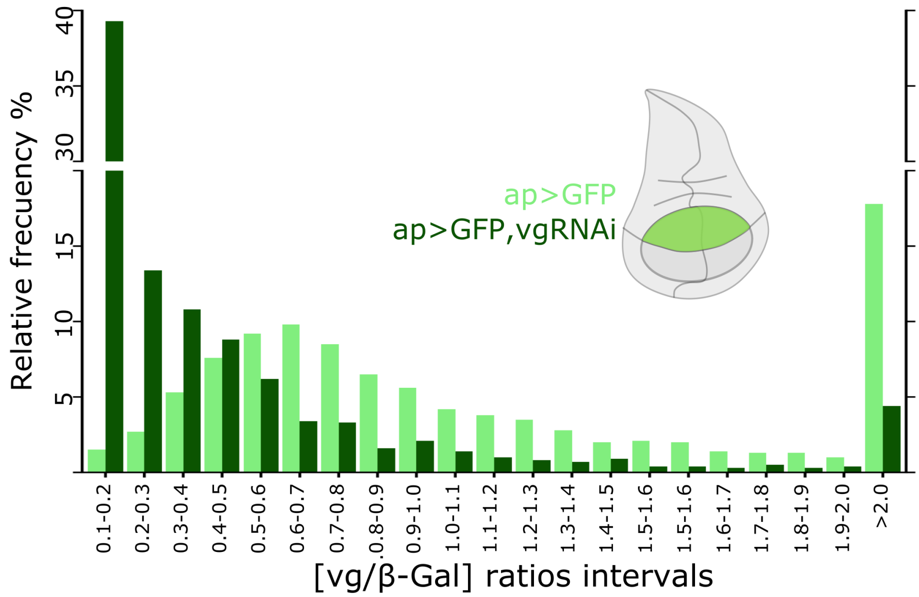

Fig. S2 (related to Fig. 3). Comparison of nuclear Vg to cytoplasmic β-Gal ratios in control (ap-Gal4, UAS-GFP) *vs.* Vg-knockdown (ap-Gal4, UAS-GFP, UAS-*vg*RNAi) cells. Distributions of nuclear Vg to cytoplasmic β-Gal ratios in cells of the dorsal compartment (light green shading in wing disc scheme) in control (light green) and *vg*RNAi-expressing cells (dark green) using the methodology of Fig. S1. The light green distribution peaks in [Vg /β-Gal] ratios higher than 2.0, whereas the dark green distribution peaks in Vg to β-Gal ratios lower than 0.2, when imaged under identical conditions.

Biology Open • Supplementary information

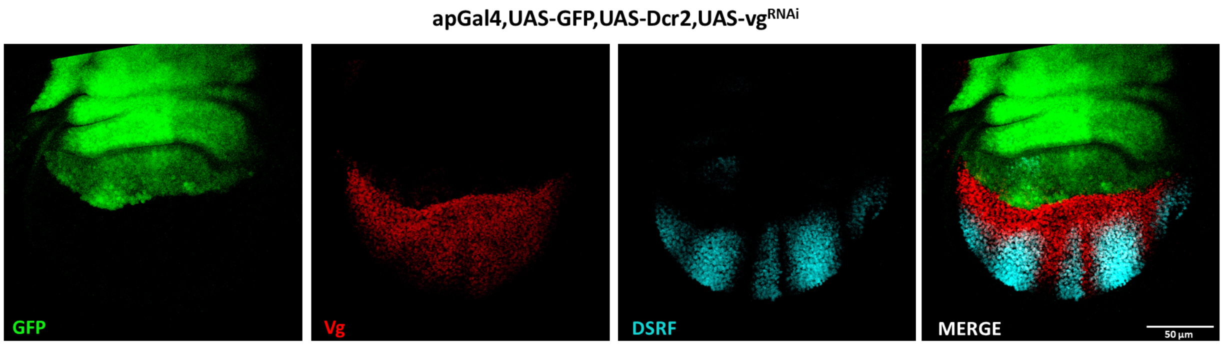

Fig. S3 (related to Fig. 3 and 4). Vg knockdown in cells of the dorsal compartment eliminates DSRF expression. Using an *ap*-Gal driver we co-expressed GFP, Dcr2 (to potentiate the RNAi effect of the RNAi), and *vg*RNAi. DRSF, the product of the Vg target gene *blistered*, is missing from the intervein regions of the dorsal compartment.

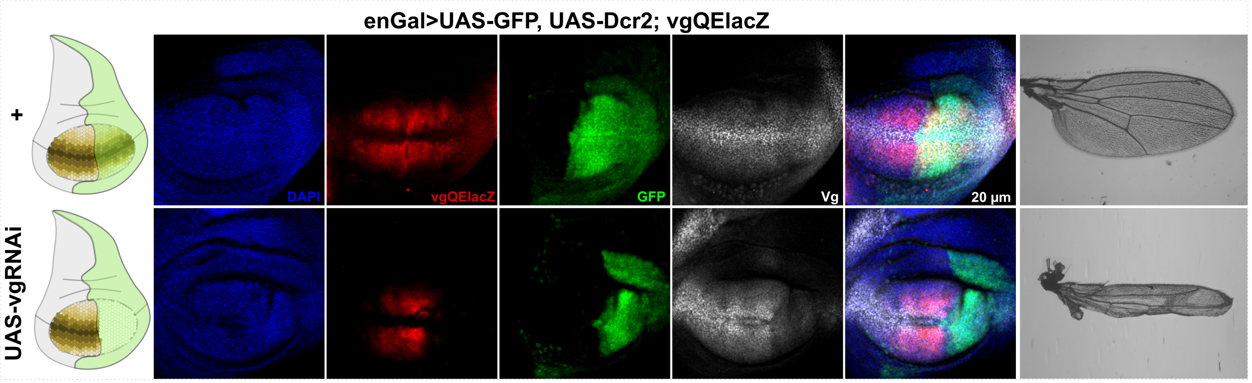

Fig. S4 (related to Fig. 3 and 4). Vg knockdown in the posterior compartment abolishes *vg*^{QE} expression and impairs wing-cell differentiation. Experiment design equivalent to that of Fig. 3 except that an *engrailed* (*en*)-Gal4 driver (instead of *ap*-Gal4 driver) is used to knock Vg expression down in the posterior compartment of the wing disc (green region in the scheme). Representative control and *vg*RNAi discs were immunostained with Vg and β-Gal antibodies (from left to right fluorescent labels are: DAPI, *vg*^{QE}lacZ, GFP, Vg and merge). In the far right column, representative adult wings of these genotypes are shown.
